# Modeling eye movement in dynamic interactive tasks for maximizing situation awareness based on Markov decision process

**DOI:** 10.1038/s41598-022-17433-3

**Published:** 2022-08-02

**Authors:** Shuo Ma, Jianbin Guo, Shengkui Zeng, Haiyang Che, Xing Pan

**Affiliations:** 1 School of Reliability and Systems Engineering, Beihang University, Beijing, 100191 China; 2 School of Automation Science and Electrical Engineering, Beihang University, Beijing, 100191 China; 3 Science and Technology On Reliability and Environmental Engineering Laboratory, Beijing, 100191 China

**Keywords:** Psychology, Human behaviour

## Abstract

For complex dynamic interactive tasks (such as aviating), operators need to continuously extract information from areas of interest (AOIs) through eye movement to maintain high level of situation awareness (SA), as failures of SA may cause task performance degradation, even system accident. Most of the current eye movement models focus on either static tasks (such as image viewing) or simple dynamic tasks (such as video watching), without considering SA. In this study, an eye movement model with the goal of maximizing SA is proposed based on Markov decision process (MDP), which is designed to describe the dynamic eye movement of experienced operators in dynamic interactive tasks. Two top-down factors, expectancy and value, are introduced into this model to represent the update probability and the importance of information in AOIs, respectively. In particular, the model regards sequence of eye fixations to different AOIs as sequential decisions to maximize the SA-related reward (value) in the context of uncertain information update (expectancy). Further, this model was validated with a flight simulation experiment. Results show that the predicted probabilities of fixation on and shift between AOIs are highly correlated ($R = 0.928$ and $R = 0.951$, respectively) with those of the experiment data.

## Introduction

Acquiring information from human system interfaces (HSIs) and the environment through eye movement is the fundamental for operators to maintain correct awareness of the system status and to make appropriate responses to the worksite situations^1^. Eye movement can be differentiated based on its goal as situation awareness (SA) driven and task performance driven, and SA is the underlying driver, especially for safety–critical systems^2^. SA is defined as ‘‘the perception of the elements in the environment within a volume of time and space, the comprehension of their meaning and the projection of their status in the near future’’^3^. Statistics show that failures of SA account for 80% of accidents attributable to human-factor causes in safety–critical industries^4^. Thus, modeling SA-driven eye movement can contribute to figuring out how SA would develop under given conditions^5^ and predicting the delay time for the establishment of SA, and then explaining the mechanism of accidents in safety–critical systems.

In recent decades, many eye movement modeling methods have been proposed for different purposes, including different task type (static or dynamic tasks), different model output (fixation probability distribution or fixation temporal sequence) and different task goal (explicit goal like maximizing behavior performance or implicit goal like maximizing SA).

For static tasks, static images were widely utilized to study eye movement in free viewing^6–13^ or visual search^14–16^ tasks. Bottom-up features (such as color, luminance and intensity) and top-down factors (such as knowledge and reward) were evaluated and combined into a master saliency map to estimate the probability of attending to a location in the image^9–11^. Further, several models have been proposed to generate fixation sequence from saliency maps by employing winner-take-all (WTA) algorithm and inhibition-of-return (IOR) scheme^9,12,13^. Although these models are very successful in predicting gaze locations in static images, they can hardly generalize to dynamic interactive tasks^17^.

For dynamic tasks, models to predict probability distribution of eye fixations were firstly developed. One representative is the SEEV model proposed by Wickens et al^18^. The SEEV model postulates the probability of attending to different AOIs in a dynamic interactive task is directly related to two bottom-up factors (salience, effort) and two top-down factors (expectancy, value). It has been validated by a series of flight simulation experiments^19,20^.

Additionally, there have been several attempts to predict fixation sequence in dynamic tasks. They can be distinguished as models without considering task goal, models with explicit goal like maximizing behavior performance, and models with implicit goal like maximizing SA.

In studies without considering task goal, video games and natural videos were widely used to predict fixation sequence by approach of machine learning. These studies segmented the video into frames and regarded each frame as a static image, with only one fixation in each frame^21–25^. In one such example, Peters and Itti^21^ recorded the eye fixation data while playing video games and learned a mapping from bottom-up and top-down feature vectors to the fixation positions for individual frames. In another example, Deng et al.^24^ used eye tracking data of experienced drivers while viewing traffic driving videos to learn a convolutional-deconvolutional neural network (CDNN), with video frames and the corresponding saliency maps constructed by the drivers’ eye tracking data as input and output of the model. The most salient region in each saliency map corresponded to the fixation position. These machine learning models are task specific, so the model have to be retrained for a new task. What is more, they are black boxes, leaving us without any conceptual understanding of how bottom-up and top-down features influence eye movement.

In studies with explicit goal, behavior performance is a dominating goal to drive eye movement in dynamic interactive tasks. Sprague, Ballard and Robinson^26^ used Markov decision process to predict human visuomotor behavior in a walking task, and demonstrated that the choice of next gaze is to maximize the reward of taking a corresponding action. Inspired by this study, Johnson et al.^27^ introduced task priority into a softmax barrier model to predict human gaze deployment in a driving task, suggesting that more attention was biased towards high-priority subtask for better task performance. In another study, Tanner and Itti^28^ incorporated goal relevance, defined to measure the degree to which an object is relevant to the task, into a top-down saliency model to predict fixation position while playing a video game, and demonstrated that more gaze was directed towards objects with higher goal relevance to obtain as much score as possible in the game.

In studies with implicit goal, SA is an underlying goal to drive eye movement. Kim and Seong^29^ proposed an eye movement model for the nuclear power plant (NPP) operators using Bayesian network. This study suggested the next AOI is selectively focused on to gain the greatest information and maximize SA. Lee and Seong^1^ incorporated factors such as working memory decay and mental model into the monitoring model in^29^. Jiang et al.^30^ proposed a Markov monitoring model for operators in NPP, suggesting the next fixation is directed to the position at which the probability of capturing attention is maximal. These models predict only a single fixation choice at a time and an entire fixation sequence through fixation-by-fixation iterations.

Available fixation sequence prediction models are suitable for simple dynamic interactive tasks but not for complex ones. A distinction between simple and complex dynamic interactive tasks can be made in terms of task complexity. Task complexity is defined as a function of the amount of information involved in the task, with a value from 0 to 1^31^. Faster pace of system dynamics generates a greater amount of information and poses a greater demand for the operator to keep following the situational changes and to make sense of the observed information. Thus, it can be inferred that a task with greater information bandwidth is more complex. For complex dynamic interactive task, operators need to continuously extract information and the experienced can plan ahead multiple-step fixation choices. While for simple ones, operators often consider only a single next gaze shift.

This study aims at proposing a computational model to predict fixation sequence in complex dynamic interactive tasks, with a basic premise that the goal of eye movement is to maximize the SA-related reward of an entire fixation sequence. Two top-down factors, expectancy and value, are introduced to describe the changing characteristics of dynamic task and the reward of acquiring information to maintain SA, respectively. Finally, the model is validated by the eye movement data derived from a representative flight simulation experiment carried out by Wickens^32^ and sponsored by a NASA project called “Human Performance Modeling”.

## Assumptions of eye movement modeling

Two assumptions are made for modeling eye movement in this paper, the details of which are explained as follows.

### Assumption 1:

Eye movement in dynamic interactive tasks can be regarded as multi-stage decisions under uncertain conditions, namely sequential decisions.

For dynamic interactive tasks, information within relevant AOIs changes uncertainly. This requires operators to continuously extract new information from AOIs through eye movement to maintain high level of SA. The deployment of fixations can be considered in the context of a sequential perception–action loop^33^. Perception is referred to fixating at one location for information to update SA, and action indicates choosing the next fixation position and then performing the fixation shift^34,35^. As the loop repeats, the choices of fixation location at different moments forms a set of sequential decisions.

To model eye movement as sequential decisions, it is necessary to analyze the dynamic nature of the interactive tasks. The dynamics reflect as the update of information within relevant AOIs. This study postulates that the update probability of information is determined by the expectancy. The expectancy of an AOI is coded by bandwidth (BW)^32^. Empirically, the higher AOI bandwidth is, the higher expectation of the operator to acquire new information and the more frequently they attend to that AOI.

### Assumption 2:

Experienced operators in dynamic interactive tasks follow an optimal policy to plan multiple fixation choices for maximizing the SA-related reward of an entire fixation sequence.

It has been widely demonstrated that eye movement shows different strategic characteristics in operators with different experience levels^36–41^. Experienced operators have clearer and more consistent scanning mode, greater scan frequency and wider scan area than novice^36–39^. Besides, several studies have demonstrated that sequential eye fixations in visual search tasks are planned ahead to maximize the reward in multiple decision steps^40,41^.

In this assumption, the SA-related reward is represented by the value of AOI. The value of an AOI to a task is the product of the task value and the relevance between that AOI and the task. In dynamic interactive tasks, multiple concurrent subtasks are usually imposed to operators. For example, pilots are required to keep on the desired flightpath and detect any off-normal event while flying. In this case, the value of one such AOI is the sum of values of that AOI to all subtasks supported by it^18^.

Based on the two assumptions, we introduce an MDP to model eye movement of experienced operators in a dynamic interactive task. The eye movement model is able to calculate the optimal policy adopted by the experienced operators for maximizing the SA-related reward. In addition, the optimal policy helps to guide fixation choices under uncertain conditions to generate fixation sequences.

## The eye movement model for dynamic interactive tasks

### The framework of the eye movement model

**Figure 1:**
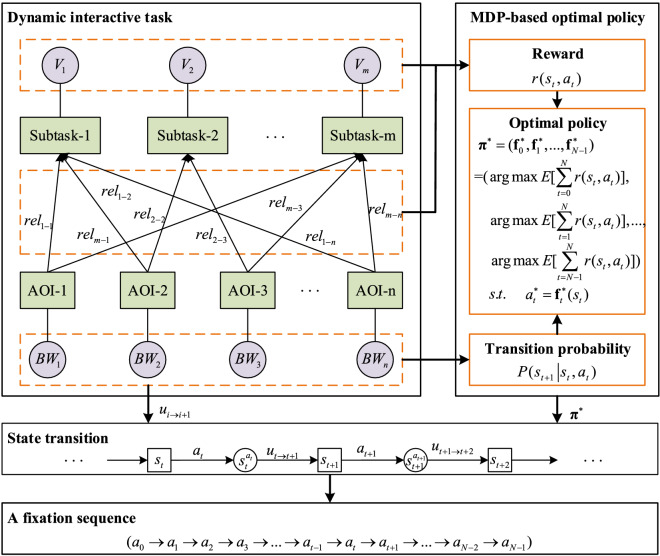
The framework of the eye movement model.

In this study, we introduce an MDP to model eye movement of experienced operators in a dynamic interactive task, with the goal of maximizing the SA-related reward. The determination of transition probability ($P(\left. {s_{t + 1} } \right|s_{t} ,a_{t} )$) and reward ($r(s_{t} ,a_{t} )$) is of crucial significance to model eye movement as an MDP. This study tries to determine these two parameters based on characteristics of the dynamic interactive task, including value of task ($V_{i}$), relevance between task and AOI ($rel_{i - j}$), and bandwidth of AOI ($BW_{i}$). The framework of the eye movement model is shown in Fig. 1.

For a specific dynamic interactive task, the modeler needs to definite the subtasks and divide the display interface into several AOIs. In addition, the modeler should also set task value for each subtask and relevance for each subtask-AOI pair according to the task goal. Then, following the framework in Fig. 1, modeling eye movement is a two-step procedure.

Firstly, obtain the MDP-based optimal policy ${{\varvec{\uppi}}}^{ * }$ for fixation choices in the dynamic interactive task. ${{\varvec{\uppi}}}^{ * }$ is a series of optimal decision rules (${\mathbf{f}}_{t}^{ * }$) which map from the current state to the best action at different decision moments and maximize the expected reward of an entire fixation sequence. It is heavily dependent on transition probability $P(\left. {s_{t + 1} } \right|s_{t} ,a_{t} )$ and reward $r(s_{t} ,a_{t} )$. The former is defined as probability of transitioning to the next SA state ($s_{t + 1}$) from the current SA state ($s_{t}$) when choosing an action ($a_{t}$). It is determined by random information update ($u_{i \to i + 1}$) between the current decision point and the next, specifically, bandwidth ($BW_{i}$) of different AOIs. And the latter is referred to value of information acquired by choosing an action when at the current SA state. It is determined by values ($V_{i}$) of subtasks as well as relevance ($rel_{i - j}$) between subtasks and that AOI only when the current SA state implies that information is unaware, otherwise it is 0. Details about modeling the transition probability and reward are described in the next section.

Secondly, use ${{\varvec{\uppi}}}^{ * }$ and AOI information update $u_{i \to i + 1}$ to generate the next state $s_{t + 1}$ from the current state $s_{t}$, and finally to obtain a fixation sequence. A fixation sequence is a series of AOIs chosen to visit when performing the dynamic interactive task. At each decision moment, which AOI to visit is determined under guidance of the optimal policy. After taking such action, the current state instantly transitions to an intermediate state $s_{t}^{{a_{t} }}$. Then the next SA state is determined by sampling information update $u_{t \to t + 1}$ according to the bandwidth of each AOI. In this way, a specific task process can be simulated to generate a specific fixation sequence.

### Obtaining MDP-based optimal policy

#### MDP-based optimal policy model

We formalize eye movement within the framework of MDP, thus the optimal policy for planning fixation choices can be represented by:

1 $${{\varvec{\uppi}}}^{ * } = ({\mathbf{f}}_{0}^{ * } ,{\mathbf{f}}_{1}^{ * } ,...,{\mathbf{f}}_{t}^{ * } ,...,{\mathbf{f}}_{N - 1}^{ * } )$$

where ${\mathbf{f}}_{t}^{ * }$ represents the optimal decision rule mapping from the current state to the best action at the decision moment $t$. The optimal decision rule maximizes the action-value function $Q_{t} (s_{t} ,a_{t} )$, which can be represented as:

2 $$\begin{gathered} {\mathbf{f}}_{t}^{ * } (s_{t} ) = \arg \max Q_{t} (s_{t} ,a_{t} ) = \arg \max E\left[\sum\limits_{i = t}^{N} {r(s_{i} ,a_{i} )} \right] \hfill \\ = \arg \max \left\{ {r(s_{t} ,a_{t} ) + \sum\limits_{{s_{t + 1} \in S}} {P(\left. {s_{t + 1} } \right|s_{t} ,a_{t} ) * Q_{t + 1} (s_{t + 1} ,a_{t + 1} )} } \right\} \hfill \\ \end{gathered}$$

$Q_{t} (s_{t} ,a_{t} )$ is defined as the expected reward of an action sequence $\left(E\left[\sum\limits_{i = t}^{N} {r\left(s_{i} ,a_{i} \right)} \right] \right)$ that begins with action $a$ taken in state $s$ at current moment $t$ and follows the optimal policy to generate subsequent actions. It consists of two parts: one is certain immediate reward $r(s_{t} ,a_{t} )$ after taking action $a$ at moment $t$; and the other is the sum of the action-values of all possible subsequent state-action pairs according to the occurrence probability $\left(\sum\limits_{{s_{t + 1} \in S}} {P(\left. {s_{t + 1} } \right|s_{t} ,a_{t} ) * Q_{t + 1} (s_{t + 1} ,a_{t + 1} )}\right)$. It can be seen that the optimal policy is able to consider how the selection of the next fixation is influenced by not only the immediate reward but the future rewards.

More detailed parameter definitions are as follows.

$t \in \left\{ {0,1,2,...N} \right\}$ is the decision moment. The time interval from one fixation choice at one decision moment to the next is called a decision period or a stage. Existing studies assume that the mean fixation interval is 300 or 500 milliseconds^26,42^, and the specific value is set by the modeler.

A state $s$ indicates the subject’s SA for the current situation in this study. At any moment, the state $s$ can be represented as:

3 $$s = \left( {i_{1} ,i_{2} ,...,i_{k} ,...,i_{n} } \right)$$

where $i_{k}$ reflects the subject's cognition of the information within the $k^{\prime}th$ AOI and $n$ is the total number of AOIs in the visual scene. $i_{k}$ is defined as:

4 $$i_{k} = \left\{ {\begin{array}{*{20}l} {0\quad \, unconscious \, of \, the \, information \, within \, the \, k^{\prime}th \, AOI} \\ {1\quad \, conscious \, of \, the \, information \, within \, the \, k^{\prime}th \, AOI} \\ \end{array} } \right.$$

Therefore, it can be inferred that the state set contains $2^{n}$ possible states.

An action $a \in \left\{ {a_{1} ,a_{2} ,...,a_{k} ,...,a_{n} } \right\}$ is one AOI in the visual scene where the gaze will be fixated next in this study.

**Figure 2:**
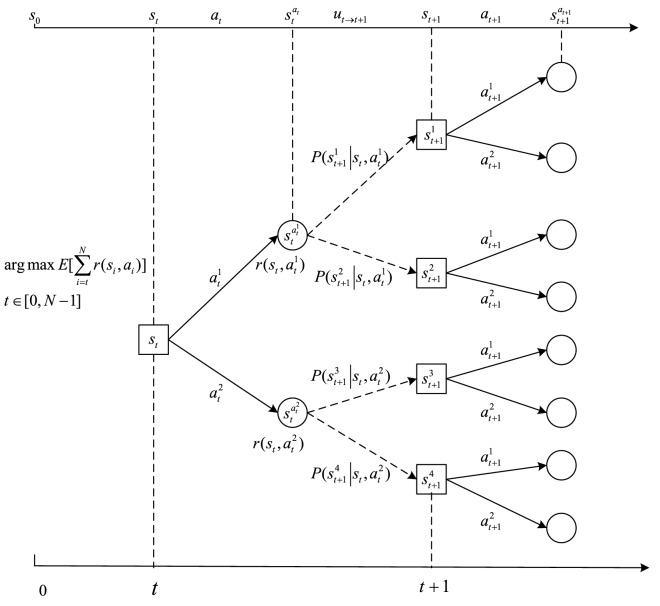
An example of the state transition process.

The state transition process, shown in Fig. 2, is depicted as follows: at some decision point $t$, the subject chooses to fixate at one AOI (taking an action $a_{t}$) and acquires the relevant information, causing the current SA state $s_{t}$ to transfer to an intermediate state $s_{t}^{{a_{t} }}$ and receiving a reward $r(s_{t} ,a_{t} )$; in the following decision period, the information within various AOIs updates randomly, which changes the SA from an intermediate state to the destination state $s_{t + 1}$ at the next decision point $t + 1$. Note that the state $s_{t + 1}$ is uncertain due to the random information update $u_{t \to t + 1}$ from $t$ to $t + 1$, and the probability of transitioning to the next state from the current state when an action has been taken is denoted as $P(\left. {s_{t + 1} } \right|s_{t} ,a_{t} )$. The modeling of the transition probability $P(\left. {s_{t + 1} } \right|s_{t} ,a_{t} )$ and the reward $r(s_{t} ,a_{t} )$ is the key of being able to model fixation sequence as an MDP, which is introduced in the following section.

#### Transition probability

To determine the transition probability $P(\left. {s_{t + 1} } \right|s_{t} ,a_{t} )$, it is necessary to pinpoint all possible next states given the current state and the action. The next states are influenced by both the action and the update of information in the visual scene, as indicated in Fig. 2.

After taking the action $a_{k}$ at moment $t$, the current state $s_{t}$ immediately transitions to an intermediate state $s_{t}^{{a_{k} }}$. The transformation process can be expressed as:

5 $$s_{t} = (i_{1t} ,i_{2t} ,...,i_{kt} ,...,i_{nt} )\mathop{\longrightarrow}\limits^{{a_{t} = a_{k} }}s_{t}^{{a_{k} }} = (i_{1t} ,i_{2t} ,...,i_{kt} = 1,...,i_{nt} )$$

which implies the $k^{\prime}th$ component of the state vector changes from 0 to 1 or maintains the value of 1 when the $k^{\prime}th$ AOI is fixated.

In the following decision period from $t$ to $t + 1$, the information updates randomly, which results in uncertain next states. Similar to the state vector, the update of information in this period can be represented as:

6 $$u_{t \to t + 1} = \left( {j_{1,t \to t + 1} ,j_{2,t \to t + 1} ,...,j_{k,t \to t + 1} ,...,j_{n,t \to t + 1} } \right)$$

where $j_{k,t \to t + 1}$ indicates the update of the information within the $k^{\prime}th$ AOI from $t$ to $t + 1$ and $n$ is the total number of AOIs in the visual scene. $j_{k,t \to t + 1}$ is defined as:

7 $$j_{k,t \to t + 1} = \left\{ {\begin{array}{*{20}l} {0\quad \, information \, within \, the \, k^{\prime}th \, AOI \, does \, not \, update \, from \, t \, to \, t + 1} \\ {1\quad \, information \, within \, the \, k^{\prime}th \, AOI \, updates \, from \, t \, to \, t + 1} \\ \end{array} } \right.$$

It can thus be seen that there are $2^{n}$ kinds of information updates. For every kind of information update, it alters the intermediate state component by component. Specifically, the $m^{\prime}th$ component of an information update $j_{m,t \to t + 1}$ acts on the $m^{\prime}th$ component of the intermediate state $i_{mt}$. The rule is as follows:

8 $$i_{mt} \mathop{\longrightarrow}\limits^{{j_{m,t \to t + 1} }}i_{m,t + 1} = \left\{ {\begin{array}{*{20}l} {i_{mt} \quad j_{m,t \to t + 1} = 0, \quad m \ne k} \\ {0\quad \, j_{m,t \to t + 1} = 1, \, m \ne k} \\ \end{array} } \right.$$

It should be noted that $i_{k,t + 1} = 1$ whether the information within the $k^{\prime}th$ AOI is updated or not, in that this AOI is continuously monitored throughout the decision period.

The transition probability depends on the information update probability that is determined by the information bandwidth of an AOI in this paper. It is hypothesized that the information update for each AOI in any decision period is independent of each other and that the information update probability of each AOI is identical in all decision periods. Then the occurrence probability for every kind of information update is calculated as:

9 $$P(u_{t \to t + 1} :s_{t} \mathop{\longrightarrow}\limits^{{a_{t} }}s_{t + 1} ) = \prod\limits_{m = 1}^{n} {P(j_{m,t \to t + 1} } )$$

where $P(u_{t \to t + 1} :s_{t} \mathop{\longrightarrow}\limits^{{a_{t} }}s_{t + 1} )$ represents the probability of one kind of information update and $P(j_{m,t \to t + 1} )$ indicates information update probability of the $m^{\prime}th$ AOI. One point should be emphasized that several kinds of information update may contribute to the same destination state given the current state and action. In this case, the transition probability $P(\left. {s_{t + 1} } \right|s_{t} ,a_{t} )$ is defined as:

10 $$P(s_{t + 1} \left| {s_{t} } \right.,a_{t} ) = \sum\limits_{h} {P(u_{{_{t \to t + 1} }}^{h} :s_{t} \mathop{\longrightarrow}\limits^{{a_{t} }}s_{t + 1} )}$$

According to the above definition, bandwidth of an AOI is key of determining information update probability and further calculating transition probability. It can be specified as^43^:

11 $$the\,bandwidth = ({{bits} \mathord{\left/ {\vphantom {{bits} {event}}} \right. \kern-\nulldelimiterspace} {event}}) \times ({{\# events} \mathord{\left/ {\vphantom {{\# events} {unit\,time}}} \right. \kern-\nulldelimiterspace} {unit\,time}})$$

which is typically defined in bits per second. ${{bits} \mathord{\left/ {\vphantom {{bits} {event}}} \right. \kern-\nulldelimiterspace} {event}}$ represents the amount of information that an event has and can be specified in the language of information theory^44^. ${{\# events} \mathord{\left/ {\vphantom {{\# events} {unit time}}} \right. \kern-\nulldelimiterspace} {unit time}}$ represents the number of events that occur in per unit of time.

Existing research divided information into discrete and continuous information^45^, and developed two corresponding methods for calculating bandwidth, respectively. For discrete information, the bandwidth is often simply expressed as events per second, such as in a driving application, the number of oncoming cars per second^46^. For continuous information, Senders proposed a method for calculating bandwidth of a pointer instrument, which is related to the change frequency of the pointer positions and the range of values and reading accuracy of the instrument^47^. Readers are referred to ^47^ for details about bandwidth calculation.

#### Reward

The reward $r(s_{t} ,a_{t} )$ means the value of information acquired by fixating at one AOI at the current state. It indicates the degree to which it is conductive of good SA state in this study. Such value is coded by the product of the task value that the AOI serves and the relevance of the AOI to the task.

The value of a task reflects its inherent importance and is represented by an integer (1, 2 3 and upward. In application, the modeler must assume some inherent task importance hierarchy. For example, the “ANCS” (Aviate, navigate, communicate, systems management) hierarchy is imposed in aviation, which indicates the task importance from highest to lowest^48^. In driving, it is assumed that lane keeping and roadway hazard detection are of greater priority (value of task = 2) than navigating (road sign detection) and in-vehicle tasks (value of task = 1)^48^.

The relevance between a task and an AOI is characterized by a value from 0 to 1. It indicates that sometimes an AOI is only partially relevant to a task. This requires the modeler to specify the degree of relevance.

For interactive tasks consisting of multiple subtasks, one AOI can be associated with several subtasks simultaneously. Then the reward for fixating at that AOI can be represented by:

12 $$r(s_{t} ,a_{t} ) = \left\{ {\begin{array}{*{20}l} {\sum\limits_{subtask} {V_{subtask} } \times rel_{subtask - AOI} \,\,\, unaware \, of \, information \, within \, the \, k^{\prime}th \, AOI} \\ {0\quad \, aware \, of \, information \, within \, the \, k^{\prime}th \, AOI} \\ \end{array} } \right.$$

where $V_{subtask}$ indicates the value of the subtask and $rel_{subtask - AOI}$ indicates the relevance between a subtask and an AOI. Note that the reward for fixating at one AOI is related to the current state. It is not 0 only when the current SA state implies information within that AOI is unaware of by the operator. It should also be emphasized that the reward for fixating at one AOI is independent of decision point, which means that the reward functions are the same at different decision points.

#### Backwards induction algorithm for optimal policy

**Figure 3:**
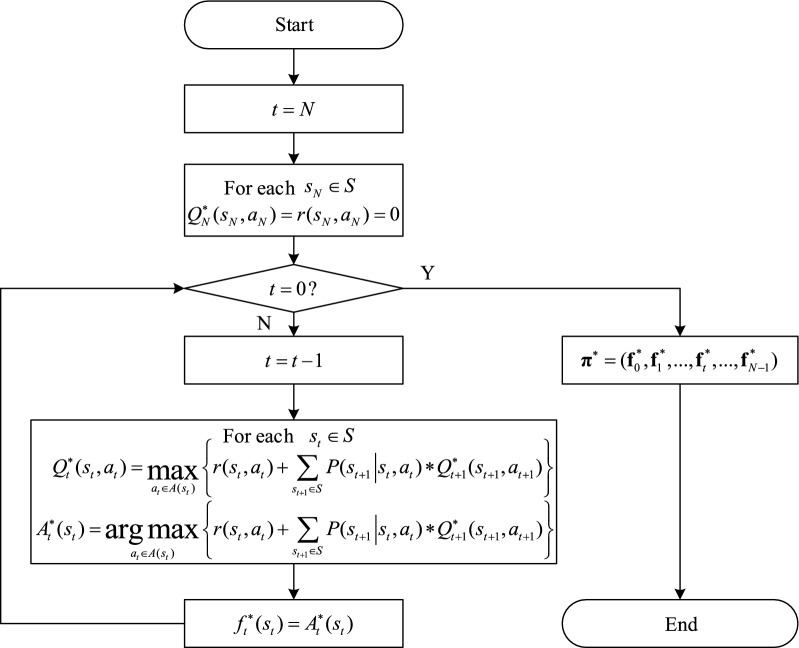
The flow chart of the backwards induction algorithm.

After defining the transition probability and the reward, we use the backwards induction algorithm to obtain the optimal policy. The flow chart of the algorithm is shown in Fig. 3.

In Step 1, the algorithm sets the decision moment as $N$ and the value function $Q_{N}^{ * } (s_{N} ,a_{N} )$ at that moment for each state as 0.

In Step 2, the algorithm needs to determine the current decision moment $t$. If $t = 0$, it indicates the optimal policy has already been obtained and the algorithm can stop; otherwise, $t$ decreases by 1 and the algorithm goes to the next step.

In Step 3, the algorithm calculates the optimal value function $Q_{t}^{ * } (s_{t} ,a_{t} )$ for each state at the decision moment $t$ according to the Bellman equation. The action that maximizes the value function for each state is the best action at that state. Note that the Bellman equation evaluates the reward of the current state, $r(s_{t} ,a_{t} )$, and the expected reward in the following states after sequentially taking the actions following the policy.

In Step 4, the algorithm returns to Step 2.

### Generating fixation sequence

**Figure 4:**
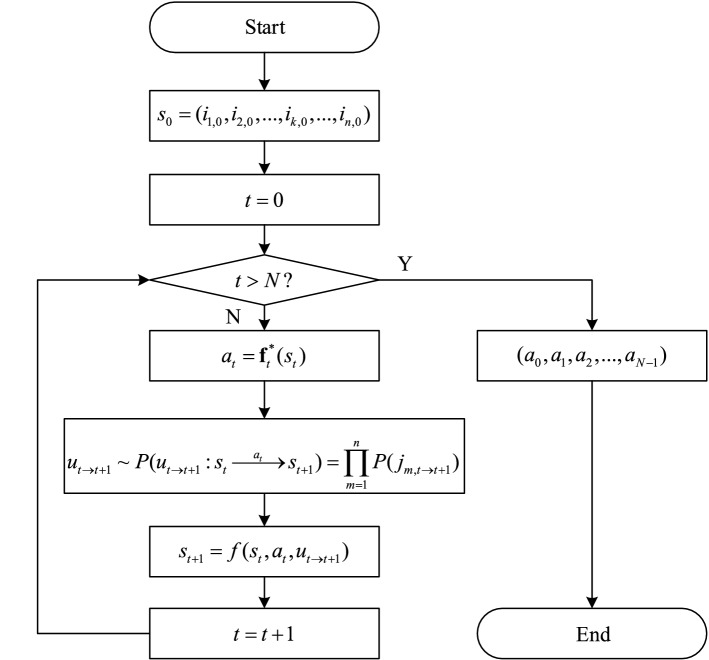
The flow chart of generating fixation sequence.

Under guidance of the optimal policy, fixation sequences can be generated by Monte Carlo simulation. The flow chart of generating fixation sequence is shown in Fig. 4.

In Step 1, an initial state $s_{0}$ at the initial moment $t = 0$ is set.

In Step 2, the current decision moment $t$ is estimated. If $t > N$, it indicates an entire fixation sequence has already been obtained and the simulation is finished; otherwise, go to the next step.

In Step 3, which AOI to fixate at given the current state $s_{t}$ is determined by the optimal decision rule ${\mathbf{f}}_{t}^{*}$ at moment $t$.

In Step 4, one kind of information update $u_{t \to t + 1}$ in this period is sampled according to the probability distribution of information update $P(u_{t \to t + 1} :s_{t} \mathop{\longrightarrow}\limits^{{a_{t} }}s_{t + 1} )$, which depends on bandwidth of each AOI $P(j_{m,t \to t + 1} )$.

In Step 5, the next state $s_{t + 1}$ is determined on the basis of the current state, the action being performed and the sampled information update.

In Step 6, the simulation moves on to the next moment $t + 1$ and returns to Step 2.

## Model validation

### Task scenario

To demonstrate the validity of the presented model, we apply it to a flight task, which is a representative dynamic interactive task and suitable for verification of the proposed model. The task scenario and experiment data used in this paper derive from a representative flight simulation experiment carried out by Wickens^32^ and sponsored by a NASA project called “Human Performance Modeling”. Details are described below.

In the flight simulation experiment, eight instrument rated pilots (6 men, 2 women) were recruited from the Institute of Aviation at the University of Illinois to fly a series of experimental curved step-down approaches to a simulated airport using a flight simulator. Pilots ranged in age from 20 to 26 years (M = 22 years) with a mean of 503 total flight hours.

**Figure 5:**
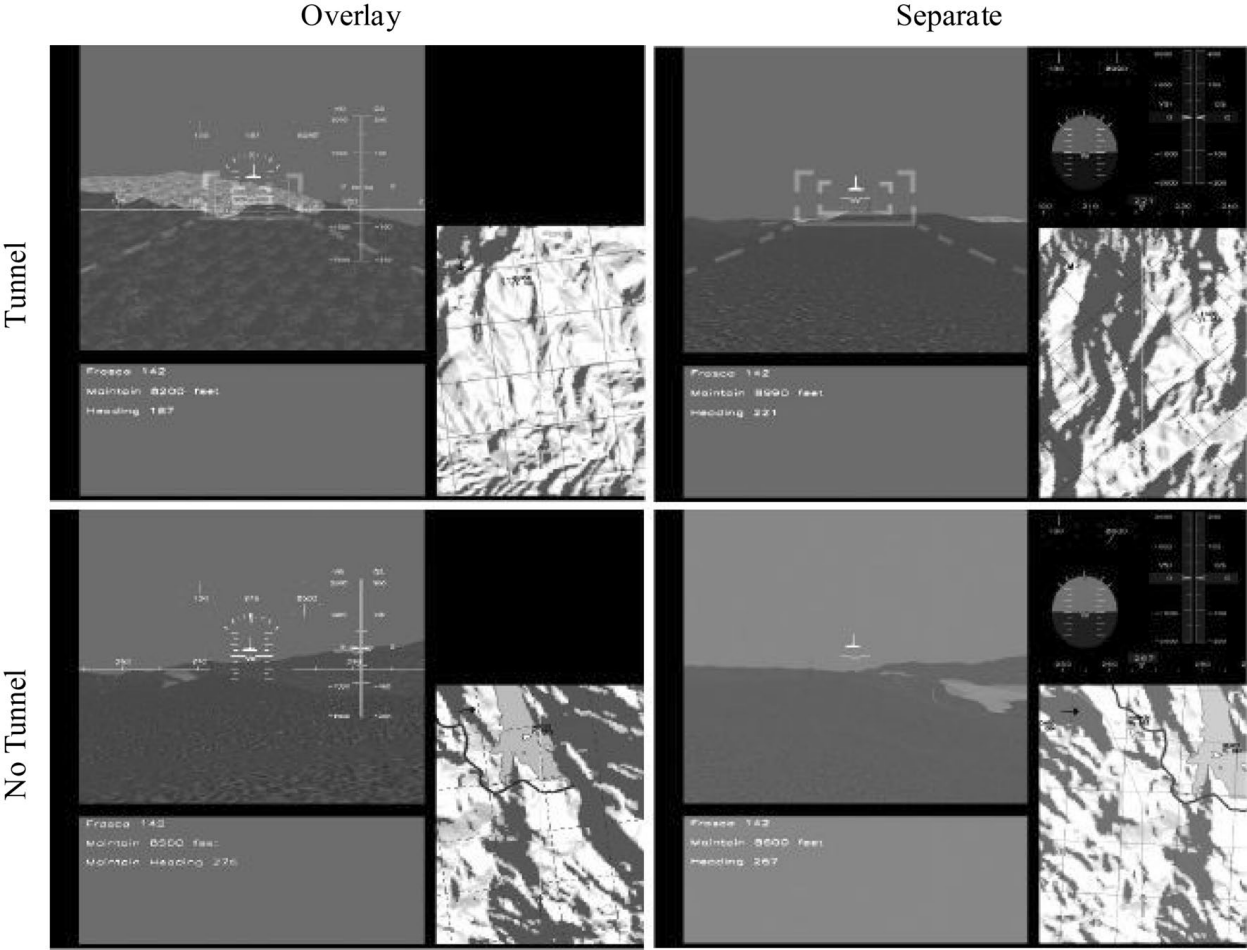
Four display suits defined by the characteristics of overlay versus separate, and tunnel versus no tunnel^32^.

The flight simulator has four versions of display suits, which are presented in a $2 \times 2$ array, as shown in Fig. 5. The two versions on the upper row contains a tunnel or “highway in the sky” to guide flightpath, while the two versions in the bottom row have no tunnel. The two display suits shown in the left column have the instrument panel overlaid on the synthetic vision system (SVS) display, while those on the right have the panel separated and positioned in the upper right corner of the suit.

In any version of display suit, there are five AOIs. The SVS display including a depiction of the terrain and the traffic visible within its field of view is located at the upper left. The instrument panel (IP) showing heading of the aircraft and vertical deviation (and deviation rate) relative to the center of the commanded flightpath is overlaid upon the SVS display or positioned at the upper right. The “datalink box” (DL) providing the guidance information such as heading and rate of climb or decent is located at the lower left. The navigation display (ND) depicting the 2D commanded flightpath and all traffic in the surrounding airspace is positioned at the lower right. Additionally, the outside world (OW) is also regarded as an AOI.

Each pilot flew two approaches with each of the four display suits, one under VMC (with the outside world visible) and the other under IMC (with the outside world obscured), each of which lasted approximately 8 min. A head-mounted eye tracker was used to track pilots’ eye movements. Both pupil and corneal reflections were sampled at 60 Hz with an accuracy of better than 1°. In each flight, pilots were instructed to conduct three parallel subtasks, including aviating (AV, controlling attitude of the plane), navigating (NAV, maintaining lateral and vertical flightpath) and hazard awareness (HAZ, noting appearance and change in terrain and traffic visible on the SVS display or the navigation display and detecting a “rogue aircraft” blimp and a runway offset visible in the outside world). Aviating has the highest priority ($V = 3$); navigating is given the second priority ($V = 2$); and hazard awareness is given the third priority ($V = 1$).

### Parameters calculation for MDP-based optimal policy model

Parameters for MDP-based optimal policy model are represented by a tuple $\left( {T,s,a,P,r} \right)$. According to the task scenario described above, the decision period in this paper is set to 500 milliseconds^42^, meaning 30 fixation samples of a 60 Hz eye tracker. Each flight contains $T = 960$ decision points. Since there are five AOIs in any version of display suit, SA state can be represented as $s = (i_{1} ,i_{2} ,i_{3} ,i_{4} ,i_{5} )$ and the action $a$ is chosen from $\{ a_{1} ,a_{2} ,a_{3} ,a_{4} ,a_{5} \}$ at any moment. The calculation of the two key parameters in this task scenario, transition probability $P$ and reward $r$, is described in detail in the following sections.

#### Calculation of transition probability

Transition probability is determined by AOI bandwidth. In this task scenario, the bandwidth of each AOI under the eight different experimental conditions is shown in Table 1. The data is derived from the original simulation experiment in^32^. It was estimated by the change frequency of variables within the AOI. Note that we set the bandwidth of IP in the four overlay conditions to 0 in this paper, because there is no information at the original position of IP.

**Table 1:** Bandwidth of AOIs across the eight experimental conditions^32^.

AOI	Experimental conditions							
	TOV	TOI	TSV	TSI	DOV	DOI	DSV	DSI
SVS	0.62	0.62	0.62	0.62	0.62	0.62	0.62	0.62
IP	0	0	0.81	0.81	0	0	0.81	0.81
ND	0.18	0.18	0.18	0.18	0.18	0.18	0.18	0.18
DL	0.05	0.05	0.05	0.05	0.05	0.05	0.05	0.05
OW	0.5	0	0.5	0	0.5	0	0.5	0

Experimental Conditions: *T* Tunnel, *D* Datalink, *O* Overlay, *S* Separate, *V* VMC, *I* IMC.

Based on Table 1, all kinds of information update and the corresponding occurrence probabilities in each experimental condition can be obtained. For brevity, the calculation of the occurrence probabilities of every kind of information update in the DSV condition is taken as an example, which is listed in Table 2. The total number of types of information update is 32. And the sum of occurrence probabilities of each information update equals to 1.

**Table 2:** The occurrence probabilities for every kind of information update.

The information update	The occurrence probability	The information update	The occurrence probability
(0,0,0,0,0)	0.0281219	(1,0,0,0,0)	0.0458831
(0,0,0,0,1)	0.0281219	(1,0,0,0,1)	0.0458831
(0,0,0,1,0)	0.0014801	(1,0,0,1,0)	0.0024149
(0,0,0,1,1)	0.0014801	(1,0,0,1,1)	0.0024149
(0,0,1,0,0)	0.0061731	(1,0,1,0,0)	0.0100719
(0,0,1,0,1)	0.0061731	(1,0,1,0,1)	0.0100719
(0,0,1,1,0)	0.0003249	(1,0,1,1,0)	0.0005301
(0,0,1,1,1)	0.0003249	(1,0,1,1,1)	0.0005301
(0,1,0,0,0)	0.1198881	(1,1,0,0,0)	0.1956069
(0,1,0,0,1)	0.1198881	(1,1,0,0,1)	0.1956069
(0,1,0,1,0)	0.0063099	(1,1,0,1,0)	0.0102951
(0,1,0,1,1)	0.0063099	(1,1,0,1,1)	0.0102951
(0,1,1,0,0)	0.0263169	(1,1,1,0,0)	0.0429381
(0,1,1,0,1)	0.0263169	(1,1,1,0,1)	0.0429381
(0,1,1,1,0)	0.0013851	(1,1,1,1,0)	0.0022599
(0,1,1,1,1)	0.0013851	(1,1,1,1,1)	0.0022599

The form of possible SA states is identical with that of the information updates, but the implications are different. According to Table 2, the three-dimensional transition probability matrix with a size of $32 \times 32 \times 5$ in the DSV condition can be acquired. We take the calculation of a row of the transition probability matrix as an example, the result of which is shown in Table 3. For brevity, the complete calculation process of the whole matrix is not described here.

**Table 3:** One example of the transition probability.

The current state	The action	The transition probability			
(0,1,1,0,1)	SVS	(1,0,0,0,0)	(1,0,0,0,1)	(1,0,1,0,0)	(1,0,1,0,1)
		0.0729	0.0729	0.3321	0.3321
		(1,1,0,0,0)	(1,1,0,0,1)	(1,1,1,0,0)	(1,1,1,0,1)
		0.0171	0.0171	0.0779	0.0779

As presented in Table 3, supposing that the current state is expressed as $(0,1,1,0,1)$ and the action taken from the current state is fixating at the first AOI (SVS), the intermediate state will be $(1,1,1,0,1)$. In consideration of all information updates and their occurrence probabilities, the possible next states and the transition probabilities can be obtained.

The calculation of the transition probability matrix is identical as that mentioned above in all conditions but for the TSV and TSI conditions. In these two conditions, the roles of the instrument panel and the tunnel located on the SVS are redundant. It means that the information within IP is also acquired when the SVS is chosen to be fixated at, but not vice versa, in that not all information within SVS is available in IP. Consequently, the calculation of the transition probability matrix in the TSV and TSI conditions should take such characteristic into account.

#### Calculation of reward

Reward for fixating at one AOI is determined by both the value of the task and the relevance of the AOI to the task. In this task scenario, values of the three subtasks, including aviating, navigating and maintaining hazard awareness, are $V = 3$, $V = 2$ and $V = 1$, respectively. The relevance of each AOI to the three subtasks under the eight conditions is illustrated in Table 4. These data are specified by the modeler and derived from^32^. Note that we set the relevance of OW to aviating and navigating in each condition to 0 in this paper, because OW is irrelevant with the two subtasks.

**Table 4:** Relevance of AOIs to subtasks^32^.

		Experimental conditions							
AOI	Task	TOV	TOI	TSV	TSI	DOV	DOI	DSV	DSI
SVS	AV	1	1	1	1	1	1	1	1
SVS	NAV	1	1	1	1	1	1	0	0
SVS	HAZ	1	1	1	1	1	1	1	1
IP	AV	0	0	0	0	0	0	0.5	0.5
IP	NAV	0	0	0.5	0.5	0	0	1	1
IP	HAZ	0	0	0	0	0	0	0	0
ND	AV	0	0	0	0	0	0	0	0
ND	NAV	0.5	0.5	0.5	0.5	1	1	1	1
ND	HAZ	0.5	0.5	0.5	0.5	0.5	0.5	0.5	0.5
DL	AV	0	0	0	0	0	0	0	0
DL	NAV	0	0	0	0	1	1	1	1
DL	HAZ	0	0	0	0	0	0	0	0
OW	AV	0	0	0	0	0	0	0	0
OW	NAV	0	0	0	0	0	0	0	0
OW	HAZ	0.5	0	0.5	0	0.5	0	0.5	0

Based on the relevance in Table 4 and the values of subtasks, the reward for fixating at one AOI can be calculated according to Eq. (12). Since it is independent of decision point, the result for any moment is the same. For lack of space, only partial reward function (for one decision point in the DSV condition) is shown in Table 5. As can be seen, the total number of possible states in each decision point is 32. At each possible state, all the five actions can be possibly selected and a corresponding reward can be obtained.

**Table 5:** The reward for one decision point in the DSV condition.

The current state	The action					The current state	The action					The current state	The action					The current state	The action				
	SVS	IP	ND	DL	OW		SVS	IP	ND	DL	OW		SVS	IP	ND	DL	OW		SVS	IP	ND	DL	OW
(0,0,0,0,0)	4	3.5	2.5	2	0.5	(0,1,0,0,0)	4	0	2.5	2	0.5	(1,0,0,0,0)	0	3.5	2.5	2	0.5	(1,1,0,0,0)	0	0	2.5	2	0.5
(0,0,0,0,1)	4	3.5	2.5	2	0	(0,1,0,0,1)	4	0	2.5	2	0	(1,0,0,0,1)	0	3.5	2.5	2	0	(1,1,0,0,1)	0	0	2.5	2	0
(0,0,0,1,0)	4	3.5	2.5	0	0.5	(0,1,0,1,0)	4	0	2.5	0	0.5	(1,0,0,1,0)	0	3.5	2.5	0	0.5	(1,1,0,1,0)	0	0	2.5	0	0.5
(0,0,0,1,1)	4	3.5	2.5	0	0	(0,1,0,1,1)	4	0	2.5	0	0	(1,0,0,1,1)	0	3.5	2.5	0	0	(1,1,0,1,1)	0	0	2.5	0	0
(0,0,1,0,0)	4	3.5	0	2	0.5	(0,1,1,0,0)	4	0	0	2	0.5	(1,0,1,0,0)	0	3.5	0	2	0.5	(1,1,1,0,0)	0	0	0	2	0.5
(0,0,1,0,1)	4	3.5	0	2	0	(0,1,1,0,1)	4	0	0	2	0	(1,0,1,0,1)	0	3.5	0	2	0	(1,1,1,0,1)	0	0	0	2	0
(0,0,1,1,0)	4	3.5	0	0	0.5	(0,1,1,1,0)	4	0	0	0	0.5	(1,0,1,1,0)	0	3.5	0	0	0.5	(1,1,1,1,0)	0	0	0	0	0.5
(0,0,1,1,1)	4	3.5	0	0	0	(0,1,1,1,1)	4	0	0	0	0	(1,0,1,1,1)	0	3.5	0	0	0	(1,1,1,1,1)	0	0	0	0	0

### Results analysis

#### The optimal policy and the fixation sequence

1. **The optimal policy**
  Given the number of decision stages, the transition probability matrix and the reward function, it is straightforward to acquire the optimal policy utilizing the backwards induction algorithm. The optimal policy in each condition is a matrix with a size of 32⨯960. Each column of the optimal policy matrix represents an optimal decision rule at one decision moment and optimal decision rules at different decision moments are the same.
  For simplicity, only the optimal decision rule at one moment in the DSV condition is presented in this section, as shown in Table 6. The column ‘the current state’ contains 32 possible states. The column 'the action' represents the optimal action that should be taken from the current state.
2. **The fixation sequence**
  Based on the optimal policy, multiple fixation sequences can be generated by setting an initial SA state and sampling information update in each period according to the bandwidth of each AOI. Each fixation sequence in each condition contains 960 choices of fixation position (AOI). An example of fixation sequence in the DSV condition is $(SVS \to IP \to DL \to SVS \to IP \to ND \to SVS \to IP \to ND \to ...)$.

**Table 6:** The optimal decision rule at one decision moment in the DSV condition.

The current state	The action	The current state	The action	The current state	The action	The current state	The action
(0,0,0,0,0)	SVS	(0,1,0,0,0)	SVS	(1,0,0,0,0)	IP	(1,1,0,0,0)	ND
(0,0,0,0,1)	SVS	(0,1,0,0,1)	SVS	(1,0,0,0,1)	IP	(1,1,0,0,1)	ND
(0,0,0,1,0)	SVS	(0,1,0,1,0)	SVS	(1,0,0,1,0)	IP	(1,1,0,1,0)	ND
(0,0,0,1,1)	SVS	(0,1,0,1,1)	SVS	(1,0,0,1,1)	IP	(1,1,0,1,1)	ND
(0,0,1,0,0)	SVS	(0,1,1,0,0)	SVS	(1,0,1,0,0)	IP	(1,1,1,0,0)	DL
(0,0,1,0,1)	SVS	(0,1,1,0,1)	SVS	(1,0,1,0,1)	IP	(1,1,1,0,1)	DL
(0,0,1,1,0)	IP	(0,1,1,1,0)	SVS	(1,0,1,1,0)	IP	(1,1,1,1,0)	OW
(0,0,1,1,1)	SVS	(0,1,1,1,1)	SVS	(1,0,1,1,1)	IP	(1,1,1,1,1)	SVS

**Figure 6:**
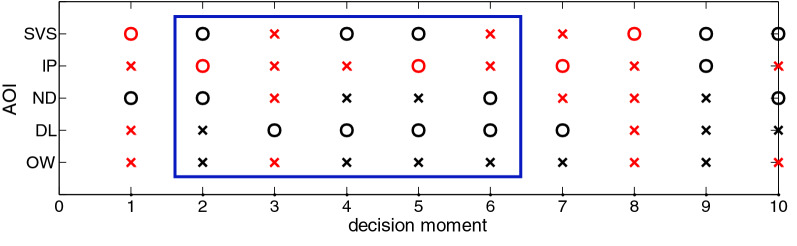
A fragment of the SA development process in the DSV condition.

On the basis of the fixation sequence, the development of SA state under given information update can be figured out. A fragment of the SA development process corresponding to the aforementioned fixation sequence in the DSV condition is shown in Fig. 6.

The horizontal axis shows decision moment, while the vertical axis represents SA corresponding to the five AOIs. The symbol “○” indicates the information in that AOI is known by the operator, while the symbol “⨯” indicates not. The red symbol means the information in that AOI has updated, while the black symbol means not. The delay time for establishing SA corresponding to one AOI can be predicted by the number of consecutive “⨯”. Taking the sub-fragment framed in blue in Fig. 6 as an example, it indicates information in ND updated in the third stage, together with information in SVS, IP and OW. The fixation choice was not to ND until the sixth decision moment, implying the delay time for noticing the updated information in ND is 1.5 s.

#### Comparison of probability of fixation on AOIs

The fixation sequence is a random series and varies with subjects and trials. Comparison of fixation sequences predicted by the model with raw eye movement data makes no sense. However, it is suggested that a random fixation sequence is dominantly constrained by the relative frequencies of fixation on AOIs^47^. That is to say, for random fixation sequences, the relative number of fixations on each AOI will converge over a sufficiently long time interval and large number of trials and can be used to validate the proposed method.

Through multiple simulations, the model can generate a set of fixation sequences. The number of fixations at each AOI was normalized within those simulated fixation sequences to estimate the probability of fixation on that AOI. The comparison of proportion of fixation on AOIs predicted by our model with experimental measuring is presented in Table 7.

**Table 7:** Predicted and experimentally observed fixation probabilities of each AOI in the eight experimental conditions.

Experimental conditions								
	TOV	TOI	TSV	TSI	DOV	DOI	DSV	DSI
**Predicted values**								
SVS	0.54	0.80	0.47	0.75	0.54	0.80	0.37	0.44
IP	0.00	0.00	0.07	0.05	0.00	0.00	0.42	0.42
ND	0.14	0.15	0.15	0.15	0.15	0.15	0.11	0.10
DL	0.05	0.05	0.05	0.05	0.05	0.05	0.04	0.04
OW	0.17	0.00	0.26	0.00	0.26	0.00	0.06	0.00
**Observed values**								
SVS	0.66	0.80	0.68	0.71	0.65	0.68	0.29	0.33
IP	0.00	0.00	0.05	0.07	0.00	0.00	0.28	0.27
ND	0.18	0.14	0.15	0.12	0.17	0.18	0.24	0.26
DL	0.03	0.04	0.04	0.04	0.09	0.11	0.11	0.09
OW	0.12	0.02	0.07	0.06	0.10	0.03	0.07	0.06

**Figure 7:**
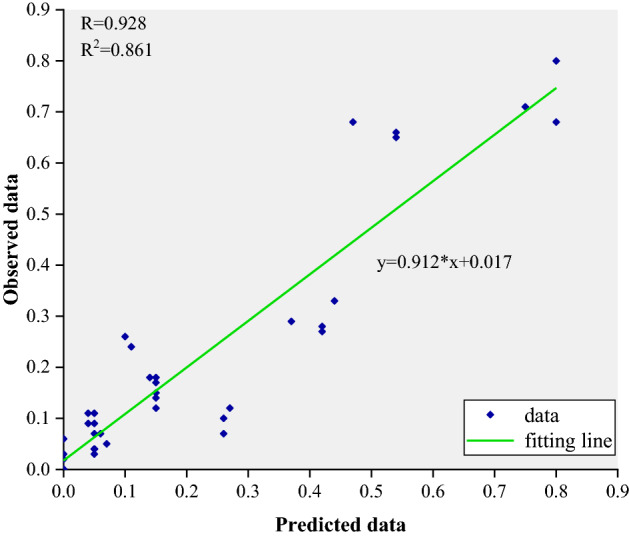
Scatter plot of predicted versus obtained fixation probability of each AOI for all conditions.

Within the first section in Table 7, the predicted fixation probability of each AOI across the eight conditions are presented. Within the second section, the experimental observed data from^32^ is presented. To demonstrate the effectiveness of the constructed model, the predicted fixation probability of each AOI was correlated against that from experiment data, as represented by the scatter plot in Fig. 7.

In Fig. 7, all 40 data points in the eight experimental conditions were correlated, with each point representing a unique combination of an AOI and a condition. As can be seen, there is a strong degree of linearity in the relation between predicted and experiment data, suggesting validation of the model. The correlation coefficient is $R = 0.928$, indicating that the model accounts for $R^{2} = 86.1\%$ of the variance in the data.

Additionally, correlation coefficients of fixation proportion on AOIs were computed within each condition, each now based upon 5 data points. The separate correlation coefficients $R$ and the $R - {\text{squared}}$ values were exhibited in Table 8.

**Table 8:** Correlation coefficients between the predicted and the observed fixation proportion on AOIs for each experimental condition.

	TOV	TOI	TSV	TSI	DOV	DOI	DSV	DSI
$R$	0.939	0.999	0.894	0.994	0.927	0.995	0.845	0.847
$R^{2}$	0.882	0.999	0.798	0.989	0.859	0.990	0.714	0.717

As is shown in Table 8, there is a strong linear correlation between the predicted and observed fixation probabilities in all conditions. It is noteworthy that the four overlay display conditions have high correlation coefficients, greater than 0.9, while the correlation coefficients of the four separate display conditions are, with only one exception, less than 0.9. This is consistent with the conclusion in^32^ that a larger distribution of information sources in different AOIs benefits a greater opportunity for individual differences in scanning strategy, hence lowering the consistency of results across pilots (lower reliability of scan data) and therefore lowering the validation correlations with model predictions.

#### Comparison of probability of fixation shift between AOIs

The probability of fixation shift between AOIs is another secondary characteristic of random fixation sequences, which tightly relates to fixation probability of AOIs^47^. To further validate this study, this statistical characteristic predicted by the proposed model is compared with experimental measuring in each condition, as shown in Table 9.

**Table 9:** Predicted and experimentally observed shift probabilities between AOIs in the eight experimental conditions.

	Predicted values								Observed values							
	TOV	TOI	TSV	TSI	DOV	DOI	DSV	DSI	TOV	TOI	TSV	TSI	DOV	DOI	DSV	DSI
SVS-SVS	0.467	0.611	0.460	0.584	0.464	0.611	0.111	0.178	0.436	0.640	0.462	0.504	0.423	0.462	0.084	0.109
SVS-IP	0.002	0.002	0.043	0.038	0.002	0.001	0.339	0.360	0.000	0.000	0.068	0.099	0.000	0.000	0.162	0.178
SVS-ND	0.261	0.291	0.179	0.232	0.270	0.291	0.268	0.307	0.238	0.224	0.204	0.170	0.221	0.245	0.139	0.172
SVS-DL	0.083	0.087	0.079	0.063	0.082	0.087	0.057	0.060	0.040	0.064	0.054	0.057	0.117	0.150	0.064	0.059
SVS-OW	0.157	0.000	0.114	0.000	0.150	0.000	0.088	0.000	0.158	0.032	0.095	0.085	0.130	0.041	0.041	0.040
IP-IP	0.000	0.000	0.000	0.000	0.000	0.000	0.000	0.000	0.000	0.000	0.003	0.005	0.000	0.000	0.078	0.073
IP-ND	0.000	0.000	0.043	0.048	0.000	0.000	0.065	0.070	0.000	0.000	0.015	0.017	0.000	0.000	0.134	0.140
IP-DL	0.000	0.000	0.007	0.011	0.000	0.000	0.015	0.011	0.000	0.000	0.004	0.006	0.000	0.000	0.062	0.049
IP-OW	0.000	0.000	0.018	0.000	0.000	0.000	0.019	0.000	0.000	0.000	0.007	0.008	0.000	0.000	0.039	0.032
ND–ND	0.000	0.000	0.000	0.000	0.000	0.000	0.000	0.000	0.032	0.020	0.023	0.014	0.029	0.032	0.058	0.068
ND-DL	0.008	0.009	0.016	0.023	0.009	0.009	0.013	0.014	0.011	0.011	0.012	0.010	0.031	0.040	0.053	0.047
ND-OW	0.017	0.000	0.029	0.000	0.018	0.000	0.021	0.000	0.043	0.006	0.021	0.014	0.034	0.011	0.034	0.031
DL-DL	0.000	0.000	0.000	0.000	0.000	0.000	0.000	0.000	0.001	0.002	0.002	0.002	0.008	0.012	0.012	0.008
DL-OW	0.006	0.000	0.013	0.000	0.004	0.000	0.004	0.000	0.007	0.002	0.006	0.005	0.018	0.007	0.015	0.011
OW-OW	0.000	0.000	0.000	0.000	0.000	0.000	0.000	0.000	0.014	0.000	0.005	0.004	0.010	0.001	0.005	0.004

Within the left section in Table 9, the predicted shift probabilities between each pair of AOIs in each condition are presented. Based on the multiple fixation sequences generated in “The optimal policy and the fixation sequence”, proportion of fixation shift between AOIs in all the sequences was estimated to represent the shift probability between AOIs.

Within the right section in Table 9, the observed probabilities of fixation shift between AOIs are presented, which were calculated on the basis of the fixation probabilities of AOIs. An approach in the literature^47^ to calculate the probability of shift between AOI *i* and AOI *j*, *P*_*ij*_, is

13 $$P_{ij} = 2P_{i} P_{j}$$

where *P*_*i*_ and *P*_*j*_ represent the probabilities of fixation on AOI *i* and AOI* j*, respectively. In particular, the probability of shift from AOI *i* to AOI *i* is *P*_*i*_^*2*^.

Additionally, the predicted shift probabilities between AOIs were correlated against those calculated from experimental data. Correlation coefficients between the two sets of shift probabilities in each experimental condition are shown in Table 10. It can be seen that there is a strong correlation between the predicted and observed shift probabilities in all conditions, further validating the effectiveness of the proposed method.

**Table 10:** Correlation coefficients between the predicted and the experimental fixation shift probabilities between AOIs for each experimental condition.

	TOV	TOI	TSV	TSI	DOV	DOI	DSV	DSI
$R$	0.992	0.991	0.991	0.975	0.992	0.983	0.807	0.876

#### Comparison with the existing models

To further validate our model, we compare the proportion of fixations on AOIs predicted by our multi-step planning model with a class of step-by-step prediction model underlain by a greedy algorithm^1,29,30^.

The model proposed in this study is capable of predicting more than the next single eye movement. It suggests that an optimal policy is followed to plan multiple fixation choices for maximizing the SA-related reward of an entire fixation sequence. The optimal policy considers how the selection of the next fixation is influenced by not only the immediate reward but the future rewards.

In contrast to our model capable of predicting multiple fixation choices, the step-by-step prediction model suggests that the next fixation is directed to the AOI at which the expected amount of information or the probability of capturing attention is maximal, underlain by a greedy algorithm. That is, these models predict only a single fixation choice at a time and an entire fixation sequence through fixation-by-fixation iterations. Based on the idea of the step-by-step prediction model, fixation sequences under the eight experimental conditions in^32^ were predicted. Statistical results about the proportion of fixation on AOIs were estimated within the simulated fixation sequences and can be seen in Table 11.

**Table 11:** Fixation probabilities of each AOI in each experimental condition predicted by the step-by-step prediction model.

AOI	Experimental conditions							
	TOV	TOI	TSV	TSI	DOV	DOI	DSV	DSI
SVS	0.51	0.98	0.48	0.95	0.48	0.92	0.46	0.45
IP	0.00	0.00	0.04	0.01	0.00	0.00	0.45	0.42
ND	0.01	0.02	0.02	0.03	0.03	0.04	0.06	0.08
DL	0.00	0.00	0.01	0.01	0.03	0.02	0.01	0.05
OW	0.48	0.00	0.45	0.00	0.46	0.00	0.02	0.00

Two sets of correlation coefficients of fixation proportion on AOIs are compared, as shown in Table 12. The first set is between data experimentally observed and predicted by our model, as same as in Table 8. And the second set is between data experimentally observed and predicted by the step-by-step prediction model.

**Table 12:** Two sets of correlation coefficients of proportion of fixations on AOIs for each experimental condition.

	TOV	TOI	TSV	TSI	DOV	DOI	DSV	DSI
$R$(predicted by our model)	0.939	0.999	0.894	0.994	0.927	0.995	0.845	0.847
$R$(predicted by the step-by-step prediction model)	0.685	0.990	0.625	0.997	0.644	0.977	0.827	0.823

Comparative result shows that our method generally outperforms the step-by-step prediction models of eye movement in a flight task. It demonstrates that our method is suitable for modeling experienced operators’ eye movement for maximizing SA in complex dynamic interactive tasks. Meanwhile, this provides quantitative support for previous empirical studies that suggest fixation sequences of experienced operators in a complex task are multi-step planning and following an optimal policy.

## Conclusions

Different from previous eye movement models focusing on static tasks or simple dynamic interactive tasks, this study suggests experienced operators are capable of planning ahead multiple fixation choices in complex dynamic interactive tasks. On this basis, a MDP model is proposed to model experienced operators’ monitoring behavior for maximizing the SA-related reward, with the deployment of fixation being regarded as sequential decisions. Two top-down factors are considered, one is expectancy coded by bandwidth to describe the update probability of the information and the other is value related to the importance of the task to represent the SA-related reward.

We applied the constructed model to a series of flight simulation tasks with eight different display suits. Statistical characteristics including probability of fixation on AOIs and probability of fixation shift between AOIs were estimated. High correlation coefficients between each statistical characteristic predicted by the model and obtained through simulation experiments verify the accuracy of the model.

Despite promising results, there are some open questions. Current study assumes SA remains constant between two fixations. Actually, limited by the capacity of memory, SA decays toward the initial state during the course of time if no more information is observed. A more plausible future extension would be taking the effect of SA decay into account to improve the eye movement model. In addition, predicting SA errors in human reliability analysis on the basis of the proposed model in this study is an another challenging topic for future research. Finally, more algorithms to predict multiple-step fixation choices can be studied to optimize time execution performance.

For possible application, the proposed method can be generalized to modeling experienced operators’ monitoring behavior for maintaining high-level SA in complex dynamic interactive tasks. Except for aviating task, the proposed method can be applied to modeling human eye movement in a car-driving task, modeling monitoring of nuclear power plant or chemical plant operators, and so on. What is more, the eye movement predicted by the model can contribute to figuring out how situation awareness would develop under given certain conditions and predicting the delay time for the establishment of SA.

## Data Availability

The data that supports the findings of this study is available from Ref 32. Request for complete result data should be addressed to Haiyang Che.
